# Transcriptional Heterogeneity Underlying Cancer Cell Migration Converges on Shared Regulatory Programs

**DOI:** 10.3390/genes17091042

**Published:** 2026-08-29

**Authors:** Ismael Ortiz, Paul V. Taufalele, Victor L. Dunagan, Samantha S. Hodge, Jing Wang, Qi Liu, Cynthia A. Reinhart-King

**Affiliations:** 1 Department of Bioengineering, Rice University, Houston, TX 77005, USA; io11@rice.edu; 2 Department of Biomedical Engineering, Vanderbilt University, Nashville, TN 37235, USA; ptaufalele@gmail.com (P.V.T.); victor.l.dunagan@vanderbilt.edu (V.L.D.); samschwager5@gmail.com (S.S.H.); 3 Department of Biostatistics, Vanderbilt University Medical Center, Nashville, TN 37232, USA; jing.wang.1@vumc.org (J.W.); qi.liu@vumc.org (Q.L.)

**Keywords:** cancer cell migration, cancer cell heterogeneity, RNA sequencing, TEAD4

## Abstract

**Background/Objectives**: Cancer cell migration is a hallmark of cancer and is associated with metastasis. While large-scale functional screens have identified regulators of migration, less is known about how intrinsic transcriptional heterogeneity drives highly migratory phenotypes within individual cancer models or whether these transcriptional changes are conserved across different models of varying tissues of origin. This study aims to define shared and cell line-specific transcriptional programs associated with cancer cell migration and analyze their relevance to patient datasets. **Methods**: Five cancer cell lines across three cancer types (breast, colorectal, and melanoma) were subjected to transwell-based migratory sorting to isolate highly and weakly migratory subpopulations. Bulk RNA sequencing, differential gene expression analysis, Gene Ontology (GO) enrichment, and upstream regulator prediction were performed. Public tumor datasets were analyzed to evaluate gene expression and its association with patient survival. **Results**: *EVA1A* was consistently upregulated in all highly migratory (HM) subpopulations. Multiple GO terms were enriched across all cell lines, often driven by distinct gene signatures, indicating convergence at the level of biological processes despite transcriptional divergence. *TEAD4* was predicted as an upstream regulator, and increased TEAD4 nuclear localization was observed in four of the five HM subpopulations. *EVA1A* and *TEAD4* expression were elevated in tumors relative to normal tissues, with cancer type-dependent survival outcomes. **Conclusions**: Migratory selection was accompanied by extensive transcriptional change within each model, yet across five models spanning three tissue types these changes converged on shared biological processes rather than shared genes. Migration-associated phenotypes may therefore be better defined by pathway-level than single-gene analyses, and the clinical relevance of regulatory nodes such as *TEAD4* appears conditional on cancer type.

## 1. Introduction

Heterogeneity is an established feature of cancer that exists among many dimensions. For example, heterogeneity is observed at the genetic, epigenetic, and tumor microenvironment levels [1]. The sources of heterogeneity include cancer stem cells, phenotypic plasticity, and clonal evolution [2]. Importantly, tumor heterogeneity may have profound implications for developing and utilizing cancer therapies. Recent work has shown that higher levels of intratumoral heterogeneity are linked with worse clinical outcomes [3]. Furthermore, several targeted therapeutics exhibit significant clinical improvements compared to previous treatments, but not every molecularly selected patient responds [4]. Thus, it is critical to investigate sources and consequences of heterogeneity.

Metastasis is the leading cause of cancer-related deaths and begins with local invasion into the surrounding stroma, making cancer cell migration an early step in this process [5,6]. To identify additional markers of cell migration, numerous groups have utilized large library genomic screens in combination with high-throughput migration assays. The majority of these studies have utilized either genome-wide or curated RNA interference screening assays followed by high-throughput migration assays to determine how disrupting particular gene expression affects migration [7,8,9,10,11,12,13,14,15,16,17,18]. However, cancer cells exhibit a high degree of heterogeneity, particularly in their migratory abilities [19]. For example, we have previously demonstrated that there exist highly and weakly migratory subpopulations within cancer cell lines [20]. Since these subpopulations may be rare but disproportionately capable of dissemination, defining underlying molecular features that distinguish these populations is of particular interest. Thus, we utilized a migratory sorting technique to obtain highly and weakly migratory cancer cell subpopulations, followed by RNA sequencing to gain further insight into heterogeneity affecting cancer cell migration ability.

Here we subjected five cancer cell lines to a transwell sorting procedure to obtain highly and weakly migratory subpopulations. To investigate molecular underpinnings of the highly migratory phenotypic subpopulations, we performed bulk RNA sequencing and differential expression analysis to determine genes and pathways associated with either phenotype. Interestingly, we found a single gene, *EVA1A*, upregulated in all five highly migratory subpopulations. Gene Ontology analysis revealed numerous biological processes regulated in all five cell lines despite substantial heterogeneity in underlying gene expression profiles. Additionally, *TEAD4* was predicted to be an activated upstream regulator in highly migratory subpopulations, and immunostaining confirmed elevated TEAD4 activity in four of five highly migratory subpopulations. Altogether, this work characterizes the transcriptional changes that accompany migratory selection within five cancer models and examines the extent to which those changes are conserved across models of differing tissue origin.

## 2. Materials and Methods

### 2.1. Cell Culture

A375 [ATCC; CRL-1619], MDA-MB-231 cells [ATCC, Manassas, VA, USA; HTB-26], MCF10CA1a cells [Barbara Ann Karmanos Cancer Institute, Detroit, MI, USA], SUM159PT cells (BioIVT; HUMANSUM-0003006), and SW480 [ATCC; CCL-228] were cultured according to the manufacturer’s instructions at 37 °C and 5% CO_2_.

### 2.2. Transwell Sorting Assay

Selected cell lines MDA-MB-231 (ATCC, Catalog No. HTB-26), MCF10CA1a (Barbara Ann Karmanos Cancer Institute, Detroit, MI, USA), and SUM159PT (BioIVT) were sorted as described by Hapach et al. SW480 (ATCC, Catalog No. CCL-228) and A375 (ATCC, Catalog No. CRL-1619) cell lines were sorted utilizing consecutive transwell assays. Desired cell populations were seeded on an 8 μm pore transwell (Corning, Corning, NY, USA) with a 1 mg/mL collagen gel with an approximate thickness of 10 μm. Cells were supplied with DMEM + 0.5% FBS and placed into a 6-well plate containing DMEM + 10% FBS. After 2 days of culture, the top reservoir was refreshed. On day 4 of culture, the media was collected, cells were washed with PBS, and trypsinized with 0.25% Trypsin-EDTA. Cells that migrated through were collected and reseeded, as were cells that did not migrate through the transwell. Consecutive transwell assays were conducted to collect the migratory cells from the initial migratory population and the non-migratory cells from the non-migratory cell population. After the invasive fraction plateaued at 25 sorts, we collected the final cells, giving us a Highly Migratory and Weakly Migratory subpopulation for each desired cell line.

### 2.3. RNA Isolation

Prior to bulk RNA sequencing, cells were cultured in tissue culture plastic 6-well plates. To isolate RNA, we utilized the QIAshredder [Qiagen, Germantown, MD, USA; 79656] and RNeasy Micro kits [Qiagen; 74004] with the on-column DNase I digest [Qiagen; 79254]. In brief, Buffer RLT, Buffer RPE, and DNase I stock solution were prepared according to the manufacturer’s instructions prior to RNA isolation. Cells were disrupted by adding 350 µL of Buffer RLT directly onto the cells in the well. The cells in Buffer RLT were then homogenized by adding the lysate directly to the QIAshredder spin column and centrifuging at max speed for 2 min. Then 350 µL of 70% ethanol was added to the lysate and applied directly to the RNeasy MinElute spin column. Then the samples were centrifuged at 10,000× *g* for 15 s to bind the sample to the spin column. The samples were washed with 350 µL of Buffer RW1 and centrifuged at 10,000× *g* for 15 s. To digest genomic DNA, 10 µL of DNase I stock solution in 70 µL Buffer RDD was added directly to each spin column and allowed to incubate for 15 min at room temperature. Then samples were washed with 350 µL Buffer RW1 and 500 µL Buffer RPE, centrifuging at 10,000× *g* for 15 s for each wash. Then a final wash with 500 µL Buffer RPE was performed at 10,000× *g* for 2 min, followed by centrifugation at 10,000× *g* for 1 min with a new empty collection tube. To elute the RNA, 35 µL of RNase-free water was added directly to the spin column and allowed to incubate at room temperature for 1 min prior to centrifugation at max speed. RNA concentrations and purity were measured via the NanoDrop instrument [Mettler Toledo; UV5 Nano], and RNA with 260/280 values > 1.7 was used for bulk RNA sequencing.

### 2.4. Bulk RNA Sequencing

RNA samples were submitted to the VANTAGE core facility at Vanderbilt University for bulk RNA sequencing. The VANTAGE core facility provided RNA quality control, stranded mRNA library preparation, and sequencing on the Illumina NovaSeq6000. Samples were utilized with an RNA integrity number equivalent (RINe) greater than 8. RNASeq libraries were prepared using 500 ng of total RNA and the NEBNext^®^ Ultra™ II RNA Library Prep [New Englad Biolabs, Ipswich, MA, USA; Cat: E7765S] per manufacturer’s instructions, with mRNA enriched via poly-A selection using oligoDT beads. The RNA was then thermally fragmented and converted to cDNA, adenylated for adaptor ligation, and PCR-amplified. The libraries were sequenced using the NovaSeq 6000 with 150 bp paired-end reads. RTA [version 2.4.11; Illumina, San Diego, CA, USA] was used for base calling, and analysis was completed using MultiQC v1.7.

### 2.5. Analysis of Cell Morphology

Cells were imaged on a Zeiss Axio Observer Z1 inverted microscope ( Carl Zeiss Microscopy GmbH, Jena, Germany) equipped with a Hamamatsu ORCA-ER (Hamamatsu Photonics, Hamamatsu, Japan) camera with a 10×/0.3 NA objective. Cell area and aspect ratios were obtained by outlining the cell body using ImageJ software (v2.16.0).

### 2.6. Bioinformatics

After adapter removal via Cutadapt [21] RNA-Seq reads were aligned to hg19 using STAR [22] and quantified by featureCounts [23]. Differential analysis was performed by DESeq2 [24]. *p*-values were adjusted for multiple testing using the Benjamini–Hochberg procedure [25], and genes with an adjusted *p* value < 0.05 and |log_2_ fold change| > 2 were considered significantly differentially expressed. Ameboid and EMT scores were computed by taking the average Z-score for manually curated lists of genes relevant to the mesenchymal-to-ameboid transition (MAT), and epithelial and mesenchymal genes, respectively (Table S1). To get the gene sets that were significantly enriched in the differentially expressed genes between highly migratory subpopulations of cancer cells compared to their weakly migratory counterparts, Gene Set Enrichment Analysis (GSEA) was run on MSigDB gene sets of hallmark, curated gene sets, regulatory target gene sets, ontology gene sets, and oncogenic signature gene sets [26,27]. Gene Ontology Biological Process (GOBP) terms were used for the cross-cell line comparisons. Over-representation analysis (ORA) of GOBP terms was performed separately in WebGestalt [28] using the significantly differentially expressed genes from each HM versus WM comparison as input and the genome as the reference gene list, with redundant terms removed. Terms with FDR < 0.05 were considered significantly over-represented. Cell-specific GO term signatures were defined by taking each GOBP term over-represented in all five cell lines and, within each cell line individually, retrieving the significantly differentially expressed genes annotated to that term.

Differential expression of *EVA1A* and *TEAD4* between normal and tumor tissue was assessed using tnmplot.com [29], which integrates transcriptomic data from TCGA, TARGET, and GTEx. The RNA-seq dataset was queried for breast, colorectal, and skin tissue, and normal versus tumor expression was compared using the Mann–Whitney U test as implemented by the platform. Sample sizes were n = 403 normal and 1097 tumors for breast, n = 315 normal and 469 tumors for colorectal, and n = 474 normal and 103 tumors for skin. Because matched adjacent normal skin is scarce in TCGA, normal skin samples were drawn predominantly from GTEx.

Patient-level expression and overall survival data for TCGA-BRCA, TCGA-COAD, TCGA-SKCM, and CPTAC-3 were obtained using Survival Genie [30]. All Cox proportional hazards models reported here were fitted in R. For each cohort, patients were dichotomized at the cohort median of *EVA1A* or *TEAD4* expression, and hazard ratios for overall survival comparing high- to low-expression groups were estimated by univariate Cox proportional hazards regression with Breslow handling of tied event times. CPTAC-3 patients were assigned to tumor types using primary-site annotations retrieved from the Genomic Data Commons API, yielding six types with sufficient events for analysis (glioblastoma, lung, head and neck, renal, pancreatic, and endometrial; n = 1151 patients with 397 deaths in total).

Two models were fitted across the full CPTAC-3 cohort. The unadjusted pooled model dichotomized all patients at a single cohort-wide median and estimated a common hazard ratio, ignoring tumor type. The stratified model dichotomized patients identically but included tumor type as a stratification factor, permitting a separate baseline hazard function for each type while constraining the expression coefficient to be shared across strata. Between-cohort heterogeneity of the individual hazard ratio estimates was quantified using Cochran’s Q statistic and the I^2^ index. Forest plots were generated in Python (v3.11.15) using Matplotlib (v3.10.9), with hazard ratios plotted on a log-scaled axis and error bars representing 95% confidence intervals.

The TEAD4 target gene signature comprised *CCN1*, *CCN2*, *ANKRD1*, *AMOTL2* and *AXL*. Each gene was standardized to a z-score across patients within a cohort, and the five z-scores were averaged to give a per-patient signature score, which was then analyzed identically to single-gene expression. In addition to median dichotomization, models were fitted with expression or signature score entered as a continuous variable standardized within cohort. Correlation between EVA1A and TEAD4 expression was assessed by Spearman rank correlation within each cohort, and across all CPTAC-3 tumor types after standardizing both genes within tumor type.

### 2.7. Transcription Factor Prediction

Upstream regulator analysis was performed using Qiagen Ingenuity Pathway Analysis (IPA, Qiagen, Redwood City, CA, USA) on the differentially expressed genes from each highly migratory versus weakly migratory comparison, analyzed separately for each cell line. For each candidate regulator, IPA reports an activation z-score, which predicts the activation state of the regulator from the direction of change in its known target genes, and a *p*-value of overlap, which tests by Fisher’s exact test whether the number of known targets among the differentially expressed genes exceeds that expected by chance. An absolute activation z-score of 2 or greater was taken as the threshold for a confident activation call (Table S2).

As an independent test of whether a single transcription factor could account for the genes shared across all five highly migratory subpopulations, EVA1A, GGT5 and TM4SF18 were input into the ‘Co-regulation’ function of the hTFtarget online resource (http://bioinfo.life.hust.edu.cn/hTFtarget), accessed on 9 May 2023 and all transcription factors with the potential to regulate at least two of the three genes were retained.

### 2.8. Quantitative Polymerase Chain Reaction (qPCR)

Previously isolated RNA was converted to cDNA via the first-strand iScript cDNA synthesis kit [Bio-Rad Laboratories, Hercules, CA, USA; 1708890]. qPCR was performed using the iQ SYBR Green Supermix [Biorad; 1708880]. The following sequences were used for qPCR: EVA1A: fwd: AGATGGCTTTGCTCAGCAACA and rev: GATGCACACGCCAGAAACAA.

### 2.9. TEAD4 Immunostaining

Cells were seeded overnight on an activated coverslip and cultured in complete medium. The next day, they were rinsed with PBS and exposed to PFA for 10 min. After 2 five-minute PBS washes, the cells were permeabilized with Triton for 10 min. Then 3 five-minute washes were performed with 0.02% Tween before blocking with a 3% BSA solution for 1 h. Finally, the rabbit anti-TEAD4 primary antibody (Abcam, Cambridge, UK; AB155244) was added at a concentration of 1:100 overnight. Three five-minute washes with a 1% BSA solution were then performed. Cells were stained with secondary antibodies AF488 phalloidin (1:500), AF568 donkey anti-rabbit (1:250), and DAPI (1:500) for one hour at room temperature in the dark. Cells were washed 3 times for 5 min with 0.02% Tween. Cells were then imaged on a Zeiss LSM800 Confocal Microscope with a 40×/1.1 N.A. long working distance water immersion objective.

## 3. Results

### 3.1. Repetitive Transwell Sorting Generates Distinct Migratory Subpopulations Across Multiple Cancer Cell Types

**Figure 1:**
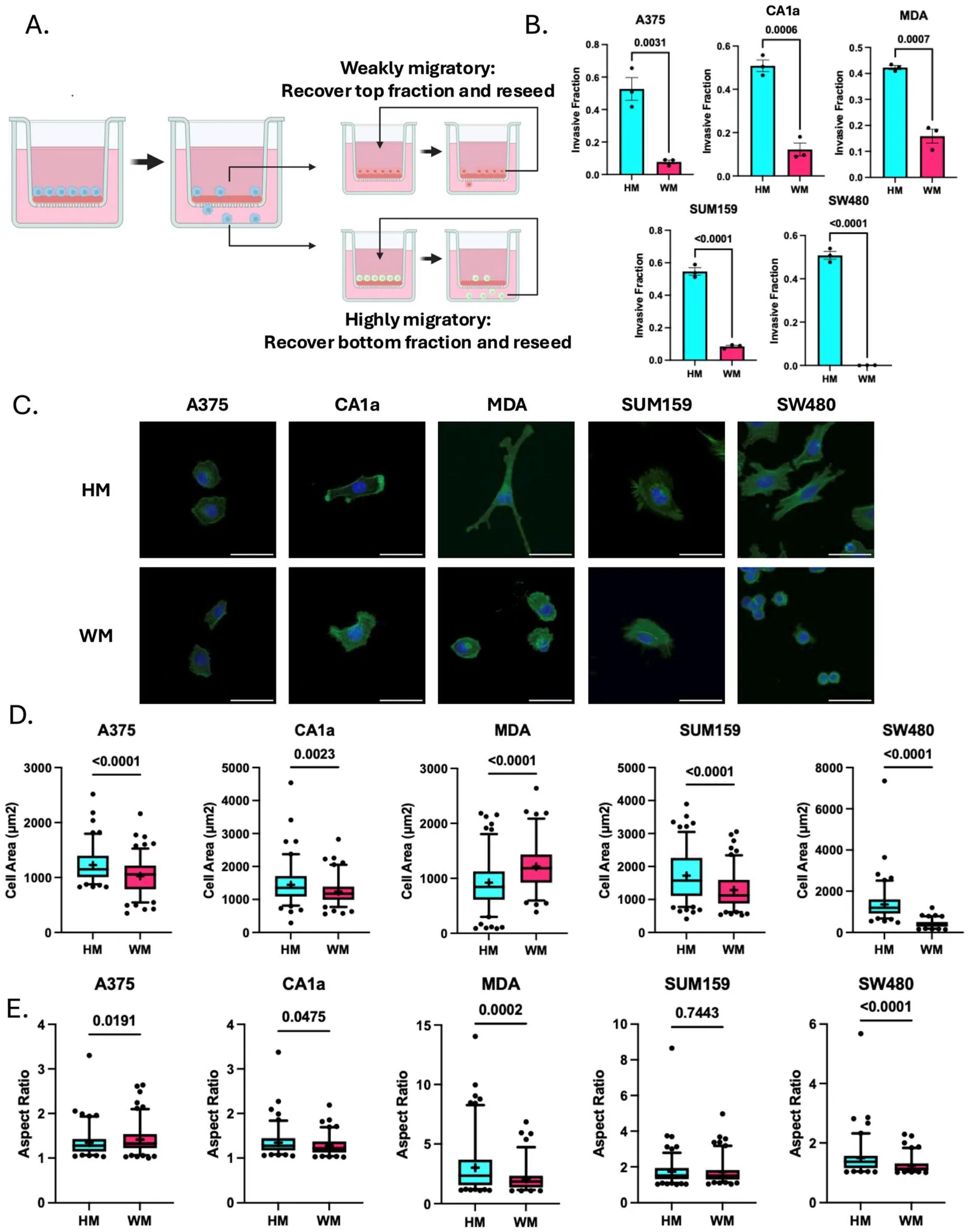
Isolation and morphological characterization of highly migratory cancer cell subpopulations. (**A**) Schematic depicting repetitive transwell sorting assay procedure. (**B**) Quantification of invasive fractions for HM and WM subpopulations. Statistical significance was assessed using an unpaired *t*-test. (**C**) Immunofluorescent images of each sorted subpopulation. DAPI = blue, actin = green. Scale bar = 50 um. (**D**) Quantification of cell area for HM and WM subpopulations. (**E**) Quantification of aspect ratio for HM and WM subpopulations. Statistical significance was assessed in D and E using a Mann–Whitney test. (**D**,**E**) N = 3, cells analyzed (HM,WM): A375 (103, 133), CA1a (113, 103), MDA (139, 97), SUM159 (139, 122), SW480 (101, 104).

To investigate the transcriptional landscapes underlying highly migratory cancer cell phenotypes, we utilized a previously described repetitive transwell sorting assay to isolate highly migratory (HM) and weakly migratory (WM) subpopulations (Figure 1A) [20]. We examined five human cancer cell lines spanning three tissue types. The breast cancer cell lines MCF10CA1a (CA1a), MDA-MB-231 (MDA), and SUM159-PT (SUM159) had previously been sorted using this approach [20], while the A375 (melanoma) and the SW480 (colorectal) cell lines were also subjected to the same sorting method (Table 1). These cell lines were selected due to their widespread use, allowing our findings to be interpreted alongside the existing literature. Additionally, each cell line exhibits documented phenotypic heterogeneity, including migratory behavior, making them strong candidates for the isolation of migratory subpopulations [20,31,32]. Finally, the three cancer types represented, one of which is of non-epithelial origin (melanoma, A375), provide a platform to ask whether transcriptional features of HM phenotypes are shared across tissue types or are specific to individual models. To confirm that the transwell sorting successfully enriched for populations with distinct, stable migratory capacities, the subpopulations obtained from all five cell lines were evaluated using a transwell invasion assay following a freeze–thaw cycle. Across all cell lines, HM populations exhibited significantly higher invasive fractions compared to their WM counterparts (Figure 1B), confirming robust separation of migratory phenotypes across all five cancer cell lines.

Representative fluorescence microscopy images further illustrate the phenotypic differences between migratory subpopulations (Figure 1C). Across multiple cell lines, HM cells frequently exhibit more elongated morphologies compared to their WM counterparts. For example, MDA and SW480 HM cells display pronounced cellular protrusions and elongated shapes, whereas WM cells appear more compact and rounded. Similar trends are observed in the CA1a and SUM159 models, although the magnitude of morphological differences varies across cell lines. The A375 subpopulations show a flipped phenotype, with the WM populations appearing more elongated than their HM counterparts. These representative images highlight the morphological heterogeneity present between migratory subpopulations.

Since cell migration is closely associated with cytoskeletal organization and cell morphology [9], we quantified cell area and aspect ratio, two morphological features associated with migratory phenotype, across all five cell lines. Interestingly, these morphological metrics did not exhibit consistent trends across models. HM populations displayed significantly larger cell areas in A375, CA1a, SUM159, and SW480 cells, whereas WM populations were significantly larger in MDA-MB-231 cells (Figure 1D). Similarly, HM cells exhibited significantly increased aspect ratios in CA1a, MDA, and SW480 cells, while the opposite trend was observed in A375 cells and no significant difference was detected in SUM159 cells (Figure 1E). Together, these results indicate that morphological characteristics vary substantially between migratory subpopulations and that phenotypic characteristics like cell morphology alone do not consistently predict migratory phenotype in this system.

### 3.2. Highly Migratory Subpopulations Exhibit Extensive but Cell Line-Specific Transcriptional Remodeling

**Figure 2:**
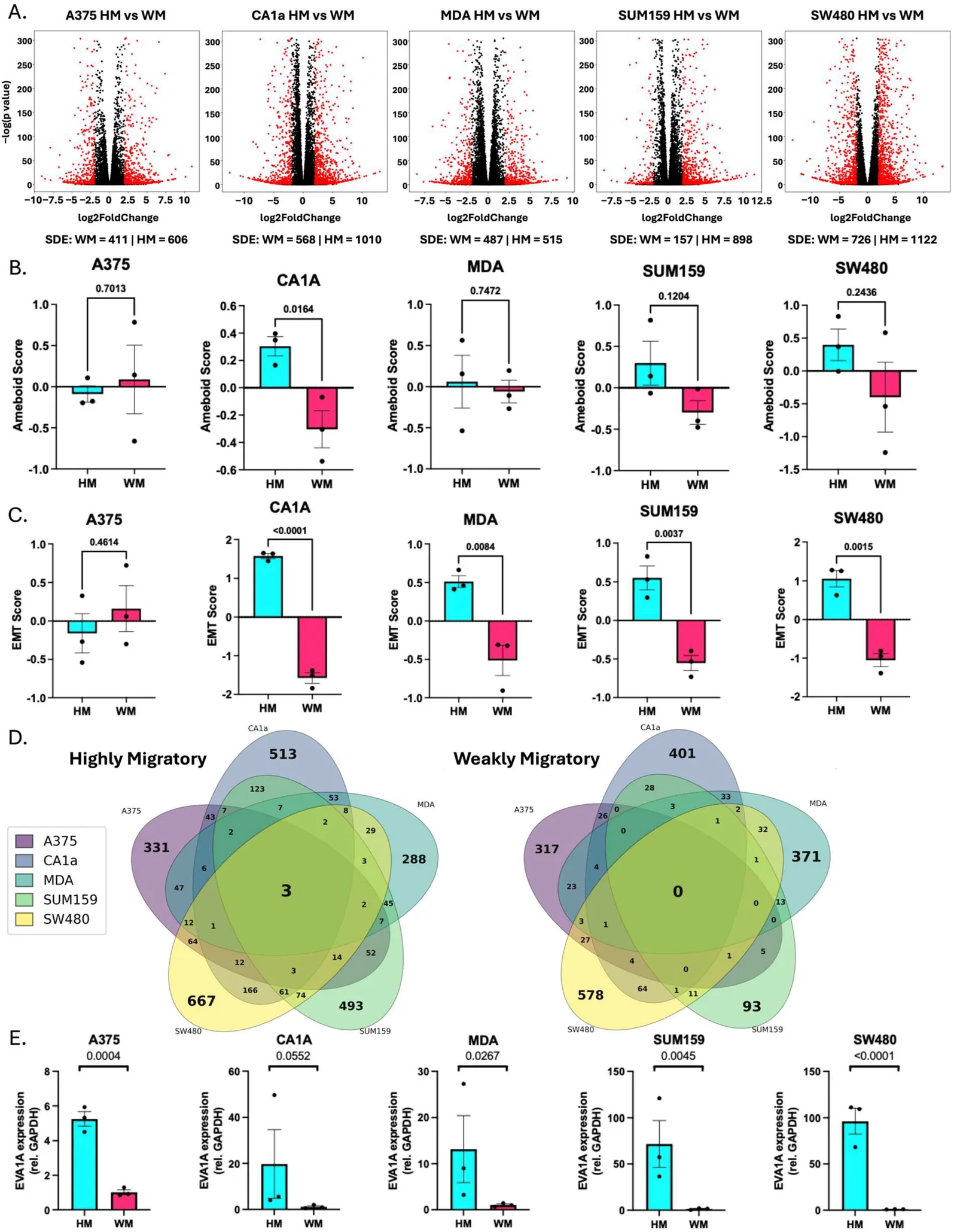
Transcriptomic analysis reveals EMT-associated signatures and limited gene overlap across migratory subpopulations. (**A**) Volcano plots representing differentially expressed genes (red dots) with a log2Foldchange of 2 and a padj of 0.05, black dots represent genes that did not meet these significance thresholds. (**B**) Quantification of ameboid scores derived from RNA sequencing data. (**C**) Quantification of EMT scores derived from RNA sequencing data. Statistical significance in B and C was assessed using an unpaired *t*-test. (**D**) Venn diagrams depicting the number of significantly differentially expressed genes that are upregulated in the HM and WM subpopulations. (**E**) Quantification of *EVA1A* expression via qPCR. Statistical significance was assessed using a lognormal *t* test.

To determine whether transcriptomic commonalities underlie migratory phenotypes, we performed bulk RNA sequencing on the HM and WM subpopulations derived from all five cancer cell lines. Differential expression analysis revealed numerous transcriptional differences between HM and WM populations within each cell line (Figure 2A). Volcano plots demonstrate widespread transcriptional changes across all models, with many genes significantly upregulated or downregulated between migratory phenotypes. Across the five cell lines, approximately 1000–1800 transcripts were identified as significantly differentially expressed. In four of the five cell lines, more genes were upregulated in HM than in WM populations (A375, 59.6%; CA1a, 64.0%; SUM159, 85.1%; SW480, 60.7% of differentially expressed genes), whereas MDA showed a balanced distribution (51.4%; Table 2). Together, these data indicate that migratory selection is accompanied by extensive transcriptional remodeling in every model examined, though the scale and directional balance of that remodeling differ between cell lines.

Ameboid and mesenchymal migration represent two well-characterized modes of cancer cell motility [33,34], and the gene expression signatures associated with these phenotypes have been previously described [35,36,37,38,39]. To evaluate whether these migration programs were enriched in HM populations, we calculated ameboid and EMT scores for each subpopulation using established transcriptional signatures [20,35]. Ameboid scores showed limited differences between migratory phenotypes across most cell lines (Figure 2B). A statistically significant increase in ameboid score was observed only in the HM population of the CA1a cells, while no significant differences were detected in the remaining four models. In contrast, EMT scores were significantly elevated in four of the five HM populations (CA1a, MDA, SUM159, and SW480) (Figure 2C). The A375 subpopulations did not display a statistically significant difference in EMT score. Together, these findings indicate that our sorting method selected for highly migratory populations enriched in EMT transcriptional programs over amoeboid-dominated programs in epithelial-based cancer cell lines.

A set of migration-associated genes shared across cancer types would represent candidate markers or therapeutic targets applicable beyond single tumor lineages, whereas limited overlap would indicate that the highly migratory phenotype is reached through different transcriptional routes in different cellular contexts. To distinguish between these possibilities, we compared genes significantly upregulated in HM and WM populations across all five cell lines (Figure 2D). Most differentially expressed genes were unique to individual cell lines. Only three genes were consistently upregulated in HM populations across all five models, while no genes were universally upregulated in WM populations. We note that these models differ in tissue of origin and genetic background in addition to the sorted migratory phenotype.

The three genes consistently upregulated in HM populations were *EVA1A*, *GGT5*, and *TM4SF18*. *EVA1A* expression, validated via quantitative PCR (qPCR), was elevated in HM populations relative to WM populations in all cell lines, consistent with the RNA sequencing results (Figure 2E). In contrast, *GGT5* and *TM4SF18* expression was not reliably detected using qPCR. Collectively, these findings indicate that migratory selection is accompanied by extensive transcriptional change within each cancer model, but that very few individual genes are shared across all five. Because the comparison within each cell line is between subpopulations that differ only in migratory selection, the genes identified in a line can be attributed to the migratory phenotype. The comparison across lines, by contrast, is confounded by differences in tissue of origin and genetic background, and the limited overlap between models should therefore not be interpreted on its own as evidence that the migratory phenotype is transcriptionally heterogeneous.

### 3.3. Highly Migratory Subpopulations Converge on Shared Biological Programs Despite Limited Gene Overlap

**Figure 3:**
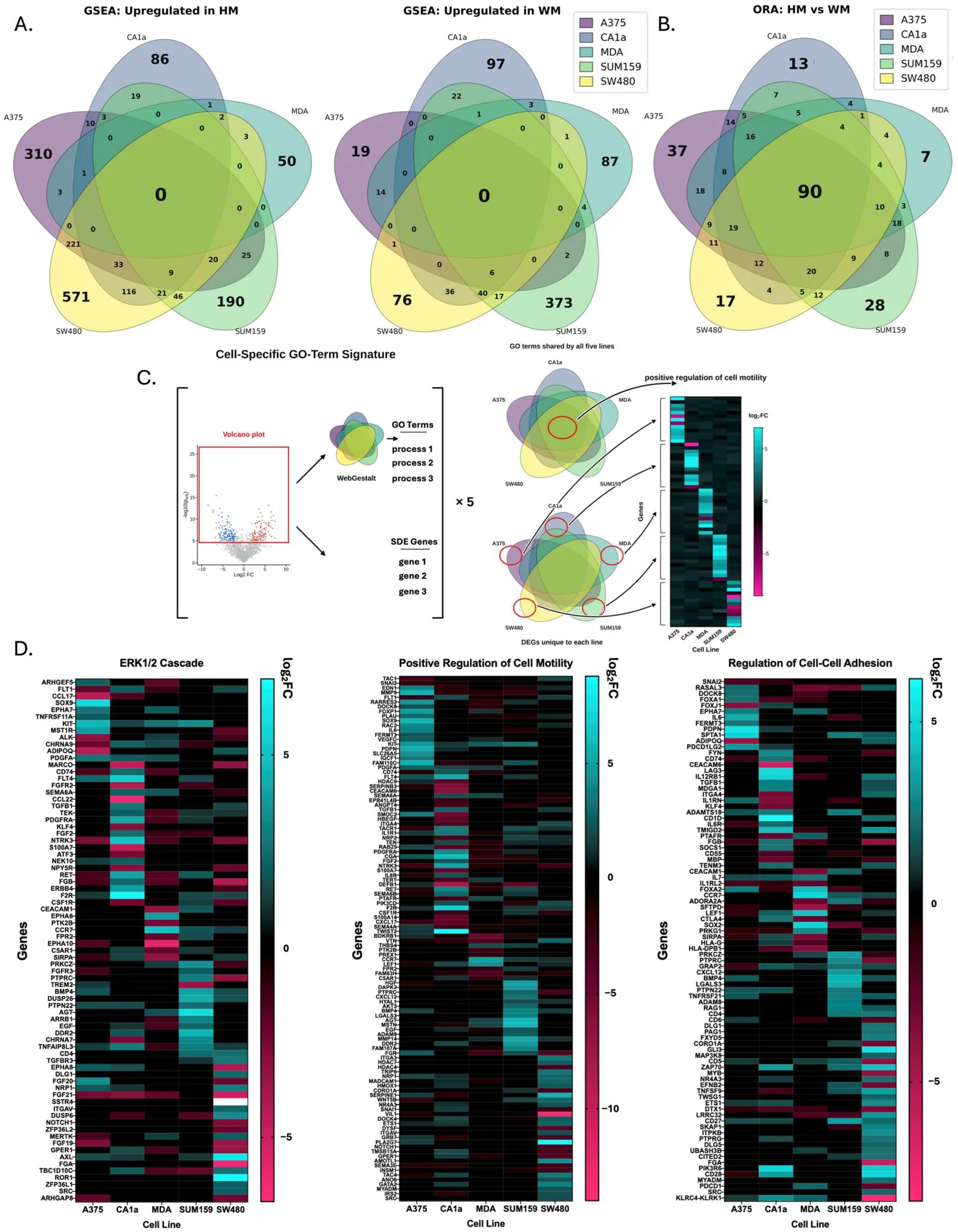
Functional enrichment converges on shared GO terms despite divergent gene-level signatures. (**A**) Venn diagrams depicting significantly enriched GO terms detected by GSEA (FDR q < 0.25) and enriched in the highly migratory (HM, **left**) or weakly migratory (WM, **right**) subpopulation of each cell line. (**B**) Venn diagram depicting GO terms significantly enriched in HM versus WM cells by over-representation analysis (ORA). (**C**) Schematic overview of cell-specific GO term signature derivation. (**D**) Representative heatmaps of cell-specific GO term signatures for ERK1/2 cascade, positive regulation of cell motility, and regulation of cell–cell adhesion; color indicates log_2_ fold change (HM/WM).

Gene Set Enrichment Analysis (GSEA) revealed numerous biological processes significantly enriched within each HM and WM subpopulation (Figure 3A). However, no individual biological process was significantly enriched across all five HM subpopulations or across all five WM subpopulations. In contrast, over-representation analysis (ORA) identified 90 biological processes significantly enriched when comparing HM and WM populations within each cell line (Figure 3B). This shared set was larger than the set of biological processes unique to any individual cell line. The discrepancy between the two approaches reflects a difference in what each test measures: GSEA evaluates whether the genes of a pre-defined set are coordinately shifted toward one end of a ranked gene list and therefore requires the set to move consistently in one direction. ORA, by contrast, is agnostic to the direction of differential expression and asks only whether more genes annotated to a given GO term appear in the input list than would be expected by chance. Thus, our results suggest that although the differentially expressed genes belong to many common biological programs, the individual genes and their direction of regulation are not shared between cancer models.

To test this directly, we repeated the ORA separately on genes upregulated in HM and WM populations (Figure S1). Only 12 biological processes were shared across all five cell lines among HM-upregulated genes, and none among the WM-upregulated genes. Because only 12 of the 90 processes remain shared once direction is considered, most shared processes are driven by genes that are upregulated in some cell lines and downregulated in others. Altogether, these results indicate that while many common biological programs are consistently associated with migratory phenotypes, the specific genes differ between cancer models in both composition and direction of regulation.

To further explore these shared programs, we defined cell-specific GO term signatures (Figure 3C). For each of the biological processes over-represented in all five cell lines, we identified the differentially expressed genes annotated to that process within each cell line individually. The resulting signatures are therefore composed of largely non-overlapping genes across the five lines, despite belonging to the same biological process. Several processes showed particularly distinct cell-specific signatures, including the ERK1/2 cascade, positive regulation of cell motility, and regulation of cell–cell adhesion (Figure 3D). Heatmap visualization of these pathways highlights the distinct gene expression patterns contributing to shared functional outcomes across the five cancer cell lines.

Together, these findings indicate that the transcriptional differences separating highly and weakly migratory subpopulations are largely cell line specific at the gene level but convergent at the level of biological process. This suggests that acquisition of a highly migratory phenotype is associated with distinct transcriptional networks identified through cancer cell line–specific gene expression programs.

### 3.4. TEAD4 Activity Is Enriched in Highly Migratory Epithelial Cancer Cells

To identify potential regulatory mechanisms underlying the transcriptional programs associated with highly migratory subpopulations, we next sought to infer upstream regulators from the differential gene expression profiles. Gene expression datasets can be used to predict upstream regulatory factors based on known causal interactions between transcription factors and their downstream targets [40]. Using Ingenuity Pathway Analysis (IPA), we performed upstream regulator analysis separately on the differentially expressed genes of each cell line. Among the candidate regulators identified, the transcription factor *TEAD4* was significantly enriched in all five models and was predicted to be activated in the highly migratory subpopulation. Four of the five models met the conventional activation threshold of z ≥ 2, with MDA-MB-231 showing a positive but sub-threshold score (Table S2).

**Figure 4:**
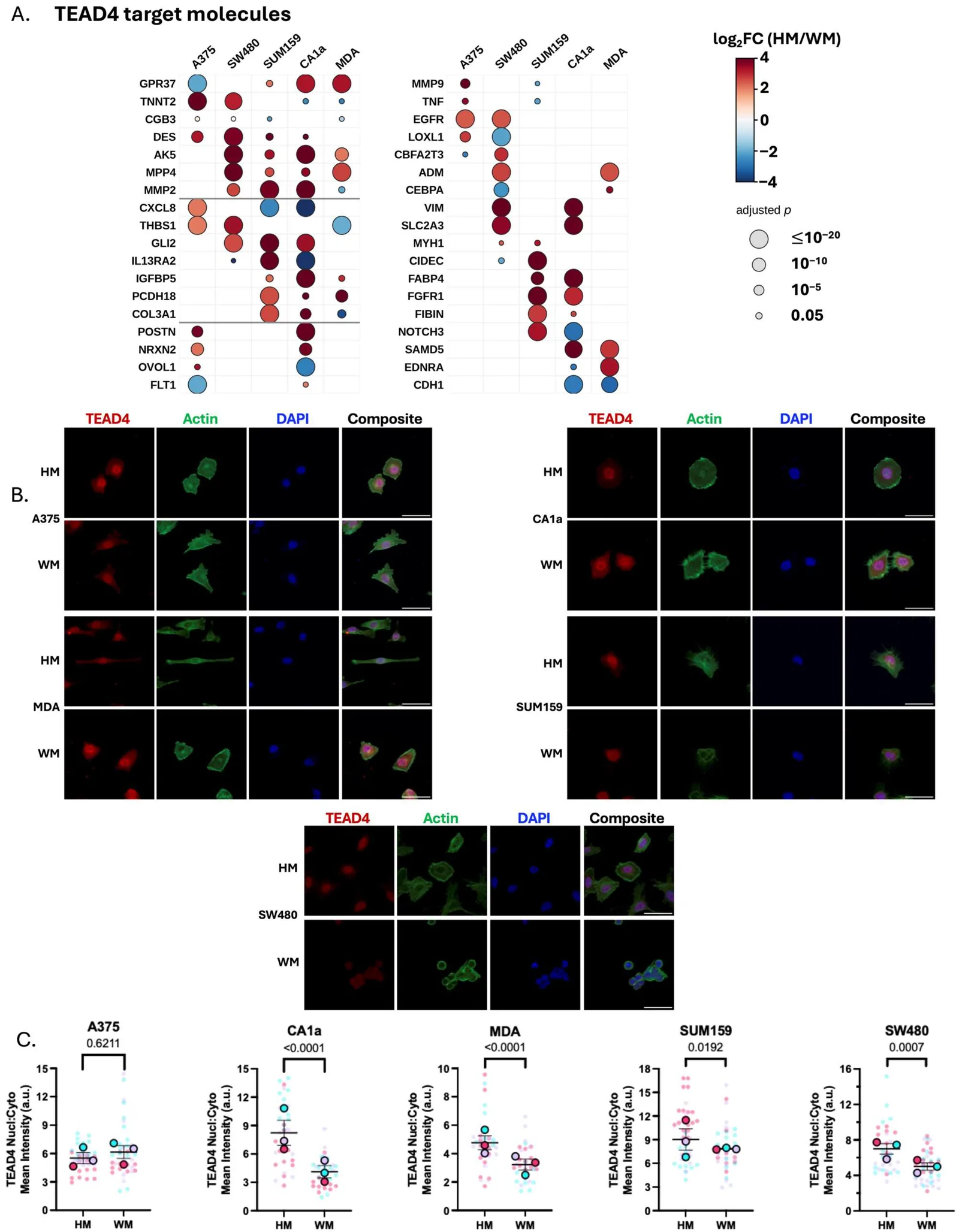
*TEAD4* regulatory networks and subcellular localization are associated with migratory phenotypes. (**A**) *TEAD4* target molecules identified by IPA upstream regulator analysis in each of the five cell lines. Each point represents a gene present in that cell line’s *TEAD4* network; color indicates the DESeq2 log_2_ fold change between highly and weakly migratory subpopulations, and point size indicates the Benjamini–Hochberg adjusted *p*-value for that change, capped at 10^−20^. Only targets present in at least two networks are shown; all 76 target molecules and their values are listed in Table S3. Genes are ordered by the number of networks in which they appear, with horizontal rules separating groups; cell lines are ordered to group genes of shared network membership and therefore do not follow the order used elsewhere. (**B**) Representative images for HM and WM subpopulations. TEAD4 = red, actin = green, DAPI = blue. Scale bar = 50 um. (**C**) Quantification of the nuclear-to-cytosolic TEAD4 mean intensity ratio in HM and WM subpopulations. Small semi-transparent points represent individual cells, color-coded by biological replicate; large, outlined points represent the mean of each independent replicate; bars indicate the grand mean ± SEM of the three replicate means. Statistical significance was assessed by a nested (hierarchical) *t*-test, N = 3 independent experiments per cell line (30–80 cells per condition in total).

The target molecules supporting these predictions were, however, largely distinct between models (Figure 4A). No target molecule was present in all five networks, seven were present in four, and 40 of the 76 targets identified appeared in only a single cell line (Table S3). Several targets present in multiple networks were regulated in opposite directions between models, including *GPR37*, which was significantly downregulated in A375 but upregulated in CA1a and MDA-MB-231, and *COL3A1*, which was upregulated in CA1a and SUM159 but downregulated in MDA-MB-231. *TEAD4* is therefore predicted to be activated in every model, but through partly non-overlapping and, in some cases, oppositely regulated sets of target genes.

To test independently whether a single transcription factor could account for the three genes upregulated across all five HM subpopulations, we queried *EVA1A*, *GGT5* and *TM4SF18* using the co-regulation function of hTFtarget. *TEAD4* was returned as a predicted regulator of two of the three genes, *EVA1A* and *TM4SF18*. That an approach independent of IPA, based only on the three shared genes rather than on the full differential expression profiles, also identified *TEAD4* supports its prediction as a common regulator across the highly migratory subpopulations.

Given these predictions, we next examined whether *TEAD4* activity was elevated in HM subpopulations. *TEAD4* transcript abundance was a poor proxy for activity across the five cell lines, as *TEAD4* was significantly differentially expressed in each line but in inconsistent directions (Figure S2, Table S4). This variability is consistent with the established regulation of *TEAD* transcriptional activity through YAP/TAZ-dependent nuclear localization rather than through *TEAD* abundance [41,42]. We therefore assessed TEAD4 activity directly by quantifying the nuclear-to-cytoplasmic (Nuc:Cyto) intensity ratio using immunofluorescence staining (Figure 4B). Representative images show TEAD4 localization (red), actin cytoskeleton staining (green), and nuclear staining with DAPI (blue) across HM and WM populations for each cell line. In several models, HM cells exhibited visibly increased nuclear TEAD4 staining compared to their WM counterparts.

Quantification of TEAD4 Nuc:Cyto intensity ratios confirmed these observations (Figure 4C). HM populations from CA1a, MDA, SUM159, and SW480 displayed significantly higher TEAD4 nuclear localization compared to WM populations. In contrast, no statistically significant difference was observed in the A375 melanoma cell line. Notably, A375 represents the only model in this study that is not derived from epithelial tissue, as melanoma originates from neural crest-derived melanocytes rather than epithelial cells. Because TEAD4 activity is frequently associated with epithelial mechanotransduction and EMT programs, the absence of differential TEAD4 activation in A375 cells may reflect lineage-specific regulatory mechanisms governing migration in melanocytic tumors.

Canonical YAP/TAZ–TEAD target genes were predominantly upregulated in HM cells across the panel (Figure S2), although their expression was not uniformly concordant with TEAD4 nuclear localization. A375 cells showed significant upregulation of all seven canonical targets despite no measurable increase in TEAD4 nuclear localization, which may reflect output sustained by other TEAD paralogues, as *TEAD1* and *TEAD3* were both upregulated in this line. Conversely, CA1a cells showed the strongest increase in TEAD4 nuclear localization but downregulation of three canonical targets. These observations indicate that canonical target gene expression alone is an imperfect readout of TEAD4 activity within individual models.

Together, these results support a model in which TEAD4 activation may function as an upstream regulator of transcriptional programs associated with highly migratory subpopulations in the four epithelial models examined, potentially contributing to the regulation of *EVA1A* expression and other genes associated with migratory phenotypes.

### 3.5. Clinical Relevance of EVA1A and TEAD4 Is Determined by Cancer-Type Context

**Figure 5:**
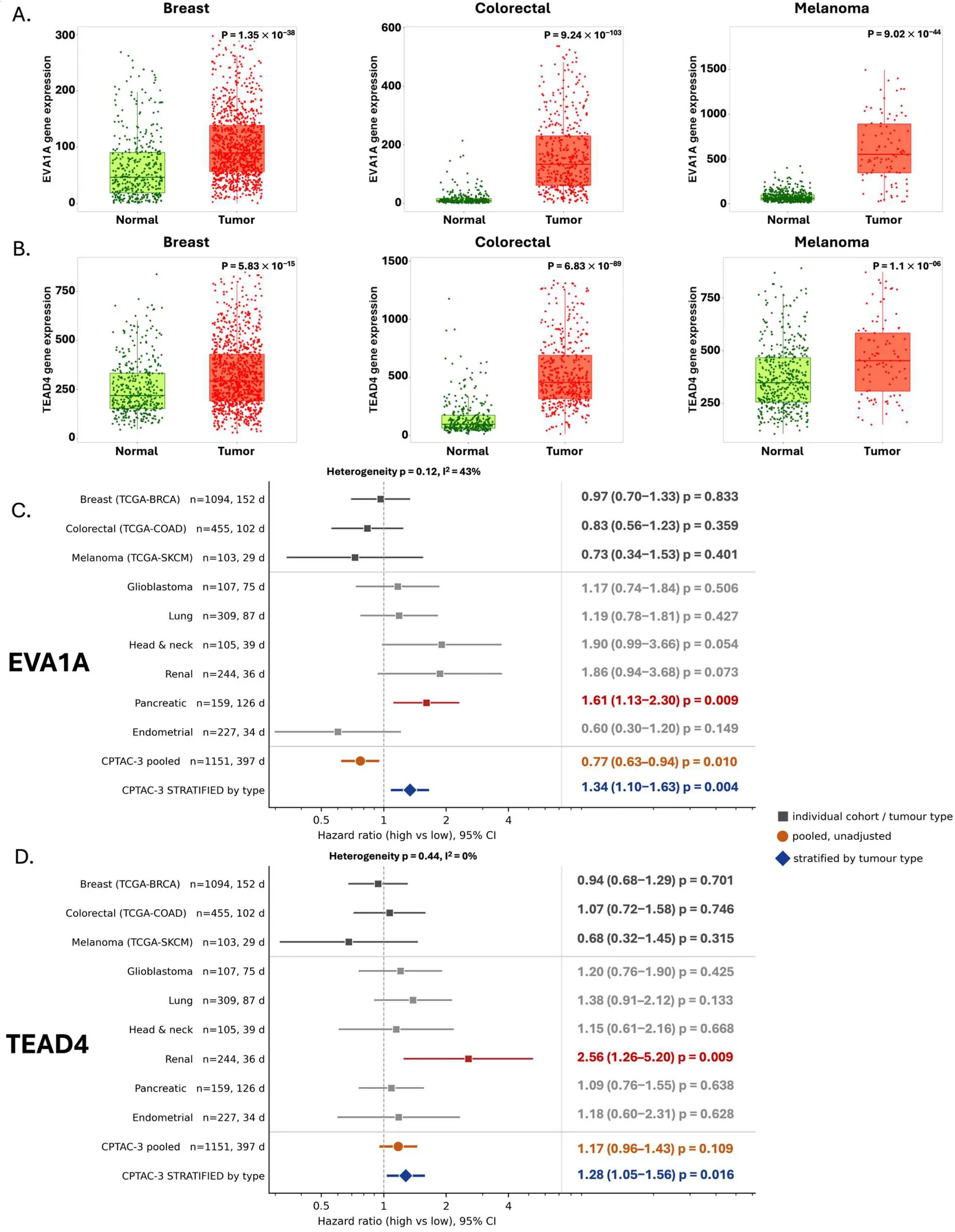
EVA1A and TEAD4 are elevated in tumor tissue and prognostic within, but not across, tumor types. (**A**,**B**) Quantification of (**A**) EVA1A and (**B**) TEAD4 expression in normal versus tumor tissue from tnmplot.com using the RNA-seq dataset. Breast, n = 403 normal and 1097 tumor; colorectal, n = 315 normal and 469 tumor; melanoma, n = 474 normal and 103 tumor. Boxes show median and interquartile range, whiskers extend to 1.5× IQR, and each point is one sample. *p*-values are from Mann–Whitney U tests. Median fold changes (tumor/normal) were 2.06, 16.22, and 9.57 for EVA1A and 1.41, 4.99, and 1.31 for TEAD4 in breast, colorectal, and melanoma, respectively. (**C**,**D**) Forest plots of hazard ratios for overall survival comparing patients with high versus low (**C**) EVA1A and (**D**) TEAD4 expression, dichotomized at the cohort median. Gray and red squares denote individual cohorts (TCGA-BRCA, TCGA-COAD, TCGA-SKCM) and individual CPTAC-3 tumor types, with red indicating *p* < 0.05; horizontal bars are 95% confidence intervals. n = patients, d = deaths. The orange circle is an unadjusted Cox model pooling all CPTAC-3 patients; the blue diamond is a Cox model of the same patients stratified by tumor type, allowing a separate baseline hazard per type. For EVA1A, pooling reverses the direction of the association (HR = 0.77, 95% CI 0.63–0.94, *p* = 0.010) relative to the stratified estimate (HR = 1.34, 95% CI 1.10–1.63, *p* = 0.004), indicating confounding by tumor-type composition; TEAD4 shows the same pattern in attenuated form (pooled HR = 1.17, 95% CI 0.96–1.43, *p* = 0.109; stratified HR = 1.28, 95% CI 1.05–1.56, *p* = 0.016). Between-cohort heterogeneity was assessed by Cochran’s Q and I^2^ (EVA1A: *p* = 0.12, I^2^ = 43%; TEAD4: *p* = 0.44, I^2^ = 0%). Dashed line, HR = 1; *x*-axis is log-scaled.

Having identified *EVA1A* and *TEAD4* as candidate regulators of the highly migratory phenotype in vitro, we tested whether there were any connections to the prognosis of patients. We first compared expression between normal and tumor tissue using TNMplot [29] for the three tissue types corresponding to our cell line models. *EVA1A* was significantly elevated in tumors relative to normal tissue in all three (Figure 5A), with median fold changes of 2.06, 16.22, and 9.57 for breast, colorectal, and melanoma, respectively. *TEAD4* was likewise significantly elevated in all three (Figure 5B) but with more modest fold changes of 1.41, 4.99, and 1.31. The smaller magnitude of the *TEAD4* shift is consistent with our in vitro observation that *TEAD4* transcript abundance changes little relative to the transcriptional output it regulates (Figure S2).

We next asked whether expression of either gene was associated with overall survival. In the three cancer type-matched TCGA cohorts, neither *EVA1A* nor *TEAD4* was significantly associated with survival when patients were dichotomized at the cohort median (Figure 5C,D; Table S5). These cohorts are, however, modestly powered for survival analysis, with 152, 102, and 29 deaths in TCGA-BRCA, TCGA-COAD, and TCGA-SKCM, respectively. To extend the analysis across a wider range of cancer types with uniformly processed data, we therefore analyzed the CPTAC-3 cohort, which comprises 1151 patients and 397 deaths across six tumor types.

Within individual CPTAC-3 tumor types, high *EVA1A* expression was associated with significantly reduced overall survival in pancreatic cancer (HR = 1.61, 95% CI 1.13–2.30, *p* = 0.009), and high *TEAD4* expression was associated with significantly reduced survival in renal cancer (HR = 2.56, 95% CI 1.26–5.20, *p* = 0.009). Most remaining tumor types showed non-significant associations in the same direction, and between-type heterogeneity was not statistically significant for either gene, indicating that the individual estimates are compatible with a common underlying effect.

Analyzing these patients as a single pooled cohort, however, produced a markedly different and misleading result. For *EVA1A*, the pooled hazard ratio indicated a protective association (HR = 0.77, 95% CI 0.63–0.94, *p* = 0.010), opposite in direction to every tumor type in which the association reached significance. Fitting a Cox model to the same patients while stratifying by tumor type, so that each type retains its own baseline hazard, reversed the estimate to the expected direction (HR = 1.34, 95% CI 1.10–1.63, *p* = 0.004). *TEAD4* showed the same pattern in attenuated form, moving from a non-significant pooled estimate (HR = 1.17, *p* = 0.109) to a significant stratified one (HR = 1.28, 95% CI 1.05–1.56, *p* = 0.016). Equivalent results were obtained by treating expression as a continuous variable rather than dichotomizing at the median (*EVA1A* stratified HR = 1.16 per SD, *p* = 0.003; TEAD4 HR = 1.12 per SD, *p* = 0.035; Table S5). This reversal arises because the six tumor types differ substantially in both baseline mortality and *EVA1A* expression, so that a single pooled median assigns patients to high- and low-expression groups that differ systematically in tumor-type composition rather than in expression alone.

Since transcription factor activity is often better reflected by the coordinated expression of downstream targets than by the transcription factor itself, we asked whether a canonical *TEAD4* target gene signature carried prognostic information where *TEAD4* expression alone did not. We scored patients using a signature defined from the established YAP/TAZ–TEAD literature rather than derived from our own data (*CCN1*, *CCN2*, *ANKRD1*, *AMOTL2* and *AXL*) [43,44]. The signature was not significantly associated with survival in any individual cohort or tumor type (Figure S3). Pooled across all CPTAC-3 patients, it appeared strongly prognostic (HR = 1.69, 95% CI 1.38–2.06, *p* = 2.8 × 10^−7^), but this association was abolished by stratification (HR = 1.07, 95% CI 0.88–1.30, *p* = 0.516) and was likewise absent when the score was treated as continuous (HR = 1.07 per SD, *p* = 0.198). The pooled signature result therefore reflects differences in baseline mortality between tumor types rather than any prognostic effect of the signature itself and illustrates the magnitude of the artifact that pooling can introduce. That a canonical, literature-defined target signature behaves this way in bulk tumor tissue is consistent with our in vitro observation that canonical target output and *TEAD4* activity are only partially concordant within individual models (Figure S2).

Finally, the relationship between the two genes was cancer-type dependent. *EVA1A* and *TEAD4* expression were positively correlated in glioblastoma, breast, and renal patients, but negatively correlated in lung carcinoma, and the correlation across all tumor types combined was indistinguishable from zero (Figure S4). Opposing within-type relationships therefore cancel when tumor types are analyzed together.

Together, these findings indicate that *EVA1A* and *TEAD4* are consistently elevated in tumor tissue and are associated with poorer overall survival within individual cancer types, but that these associations are obscured, and for *EVA1A* inverted, when patients from different tumor types are analyzed as a single cohort. The prognostic value of a given transcript is therefore conditional on tumor type rather than uniform across cancers. This parallels what we observe in our cell line models, where subpopulations from different cancer types converge on shared biological programs while differing in the specific genes through which those programs are engaged. It also suggests that the clinical relevance of a regulatory node such as *TEAD4* is more likely to be captured by measures of its activity within a defined disease context than by a fixed panel of target genes applied across cancers.

## 4. Discussion

Taken together, our findings indicate that across five cancer models of differing tissue origins, transcriptional changes accompanying migratory selection are largely model-specific at the gene level yet converge on shared biological processes related to cell motility, signaling, and cytoskeletal regulation.

Two levels of variation should be distinguished in interpreting these data. Within each model, repeated transwell sorting generated isogenic subpopulations differing only by migratory selection, and the transcriptional differences identified within a line can be attributed to the migratory phenotype. Between models, tissue of origin, lineage, and genetic background differ in addition to migratory phenotype, so comparison of the five gene lists reflects both sources of variation. Although all five HM subpopulations were obtained through the same repetitive transwell sorting assay and displayed similar migratory ability, they varied substantially in morphology, transcriptional profile, and migration-associated gene signature. Cell morphology measurements revealed no consistent trend in cell size or aspect ratio, and transcriptomic analyses identified approximately 1000–1800 differentially expressed genes between HM and WM populations in each cell line. Despite this transcriptional diversity, functional analyses revealed that migratory subpopulations consistently converged on shared biological processes across cancer types. These findings suggest that highly migratory cancer phenotypes can arise through diverse transcriptional programs that ultimately converge on common functional pathways.

In cancer, EMT is frequently linked to highly migratory phenotypes. Four of the five HM subpopulations displayed higher EMT scores compared to their WM counterparts. In addition to transcriptional changes, EMT is associated with morphological alterations, including increased cellular elongation and higher aspect ratios. Only three out of the five HM subpopulations had significantly higher aspect ratios compared to their WM counterparts. The SUM159 cells displayed no significant differences in aspect ratio, while the A375 cells displayed higher aspect ratios in the WM subpopulations. The A375 subpopulations also did not exhibit significantly different EMT scores, which may explain why the subpopulation aspect ratios do not follow the expected trend associated with EMT. However, the SUM159 cells had higher EMT scores but did not have significantly higher aspect ratios. These observations suggest that transcriptional EMT programs do not always translate directly into morphological changes and that the relationship between EMT status and cell morphology may vary depending on cellular context.

Given that migratory phenotypes were not consistently explained by morphological features alone, we next examined the underlying transcriptional programs associated with these subpopulations. We observed only a small number of genes consistently upregulated across all five HM subpopulations. This limited overlap may partly reflect lineage and genetic differences between models, which are known to influence global gene expression patterns [45,46,47]. In addition, a gene central to migration in one lineage may be unexpressed in another. Despite this heterogeneity at the gene level, our gene ontology and upstream regulator analyses revealed convergence at the pathway level, with numerous biological processes shared across all five cancer cell lines. Importantly, these shared biological programs were frequently driven by distinct sets of differentially expressed genes in each model, indicating that different cancer cell types may activate similar functional pathways through alternative transcriptional networks. Together, these findings suggest that highly migratory cancer phenotypes can arise through distinct regulatory architectures that converge on common migration-associated biological programs.

*EVA1A* was upregulated in all five HM subpopulations relative to their WM counterparts. EVA1A is associated with the lysosome and endoplasmic reticulum and has been implicated in the regulation of autophagy and apoptosis [48]. Prior studies have reported context-dependent effects of EVA1A in cancer cell migration. Increased EVA1A expression has been associated with decreased migration in hepatocellular carcinoma and breast cancer cell lines [49,50,51], but has also been linked to increased migration in human aortic endothelial cells and papillary thyroid cancer cells [52,53]. In our study, we observed increased *EVA1A* expression associated with higher migratory ability in three breast cancer cell lines (MDA-MB-231, MCF10A-CA1a, SUM159-PT), while previous work observed increased EVA1A expression associated with lower migratory ability in MDA-MB-231, MDA-MB-468, and MCF-7 breast cancer cell lines [49,50]. These discrepancies likely reflect the context-dependent effects of EVA1A across different cellular environments and signaling conditions. For example, Zhen et al. treated breast cancer cell lines with flubendazole and observed increased autophagic cell death alongside increased EVA1A expression and decreased migration [49]. Thus, the flubendazole treatment likely influences additional signaling pathways contributing to the decrease in migration. Altogether, this suggests EVA1A is likely not a universal marker of enhanced migration ability and remains dependent upon cell-, cancer-, and environment-specific contexts.

Prior studies suggest that EVA1A has context-dependent roles in cancer progression, with both tumor-promoting and tumor-suppressive effects reported across cancer types [48]. In our study, *EVA1A* expression was elevated in tumor relative to normal tissue in breast, colorectal, and skin cancers. Its association with survival, however, depended strongly on the cancer type considered. *EVA1A* was not significantly associated with survival in any of the three cancer-type-matched TCGA cohorts, and among the CPTAC-3 tumor types it was significantly associated with reduced survival only in pancreatic cancer. Analyzing all CPTAC-3 patients as a single pooled cohort produced an apparently protective association that reversed direction once tumor type was accounted for, illustrating how readily pan-cancer survival analyses can be confounded by differences in baseline mortality between cancer types. These findings demonstrate that the relationship between *EVA1a*, cellular migration, and clinical outcomes is strongly context-dependent. Cell migration is a key step in the metastatic cascade, but increased migration does not necessarily translate to worse clinical outcomes. Consistent with this, we have recently demonstrated that increased migration does not necessarily correlate with increased metastasis using breast cancer HM and WM subpopulations [20]. Together, these observations indicate that cellular migration alone is not necessarily predictive of metastatic potential or patient survival.

To identify potential regulatory drivers of these shared programs, we utilized Qiagen IPA analysis to infer upstream regulators and identified *TEAD4*, a transcription factor with canonical roles in the Hippo signaling pathway [54]. We further confirmed increased *TEAD4* activity in four of the five HM subpopulations through quantification of TEAD4 nuclear-to-cytosolic localization, with the A375 melanoma cell line being the only model that did not show increased TEAD4 nuclear localization in HM cells. Notably, A375 is the only non-epithelial cancer model examined. Because TEAD4 activity is frequently associated with epithelial mechanotransduction and EMT-associated transcriptional programs, the absence of differential TEAD4 activation in A375 cells may reflect lineage-specific regulatory mechanisms governing migration in melanocytic tumors. In the context of cancer, prior work has demonstrated that TEAD4 can promote EMT in colorectal and head and neck squamous cell carcinoma and contribute to metastasis in gastric, breast, lung, and colorectal cancers [54]. Consistent with studies demonstrating that TEAD4 activity promotes cancer cell motility, four of the five HM subpopulations exhibited both significantly higher EMT scores and increased TEAD4 nuclear localization [55].

Several limitations should be noted. The five models used here differ simultaneously in migratory phenotype, tissue of origin, and genetic background. This design enables identification of migration-associated transcriptional changes within each model but limits our ability to distinguish the effects of migratory selection from cellular background across models. The limited gene-level overlap may therefore reflect context-dependent migratory programs, lineage-specific transcriptional differences, or both. Accordingly, we describe convergence at the level of biological processes as a property of the five models examined rather than as evidence of a universal migratory transcriptional program. Distinguishing these variables will require multiple independently sorted subpopulations derived from individual lineages, or a substantially larger panel of models spanning multiple tissue types. In addition, the survival analyses presented here rely on publicly available bulk transcriptomic cohorts, where expression reflects both tumoral and stromal/immune compartments. The number of deaths within individual tumor types is also limited; therefore, non-significant associations should not be interpreted as evidence of absence.

The convergence on shared biological processes that we identified may be facilitated by shared regulatory nodes such as TEAD4, which exhibited increased nuclear localization observed in four of the five HM subpopulations. In patient tumors, *TEAD4* expression was associated with poorer overall survival within individual cancer types, though not when cancer types were pooled. By contrast, a canonical *TEAD4* target gene signature carried no prognostic information once cancer type was accounted for. This dissociation between the regulator and its canonical output parallels what we observe in vitro, where TEAD4 nuclear localization and canonical target gene expression were only partially concordant across the five models. It also suggests that a fixed panel of target genes is unlikely to serve as a transferable readout of pathway activity in bulk tumor tissue, where the expression of mechanoresponsive targets such as *CCN1* and *CCN2* reflects stromal and extracellular matrix content in addition to tumor cell state. Together, these findings highlight the importance of evaluating regulatory node activity within defined disease contexts, rather than applying single genes or fixed gene signatures uniformly across cancer types. Regulatory nodes such as *TEAD4* may therefore provide a framework for identifying shared vulnerabilities underlying complex phenotypes despite substantial gene-level heterogeneity.

## Figures and Tables

**Table 1:** Information on cancer cell lines used in this study.

Cell Line	Abbreviation	Cancer Type	Lineage	Migratory Sorting
**A375**	A375	Malignant melanoma	Neural crest-derived, non-epithelial	In this study
**MCF10aCA1a**	CA1a	Breast adenocarcinoma	Epithelial	Previously sorted
**MDA-MB-231**	MDA	Breast adenocarcinoma	Epithelial	Previously sorted
**SUM159-PT**	SUM159	Breast pleomorphic carcinoma	Epithelial	Previously sorted
**SW480**	SW480	Colorectal adenocarcinoma	Epithelial	In this study

**Table 2:** Significantly differentially expressed (SDE) genes between HM and WM subpopulations of each cell line. Genes were considered significantly differentially expressed at an adjusted *p*-value < 0.05 and log_2_ fold change > 2. SDE, significantly differentially expressed.

Cell Line	Total SDE	Up in HM	Up in WM	% Up in HM
**A375**	1017	606	411	59.6%
**CA1a**	1578	1010	568	64.0%
**MDA**	1002	515	487	51.4%
**SUM159**	1055	898	157	85.1%
**SW480**	1848	1122	726	60.7%

## Data Availability

The data will be made available upon reasonable request.

## Supplementary Materials

The following supporting information can be downloaded at https://www.mdpi.com/article/10.3390/genes17091042/s1, Figure S1: Direction-specific over-representation analysis of GO Biological Process terms in highly and weakly migratory subpopulations; Table S1: Gene lists used to calculate ameboid and EMT scores; Table S2: Ingenuity Pathway Analysis upstream regulator results for TEAD4 in each cell line; Table S3: TEAD4 target molecules identified by Ingenuity Pathway Analysis in each cell line; Figure S2: TEAD4 transcript is not consistently altered across cell lines, whereas canonical YAP/TAZ–TEAD target genes are upregulated in highly migratory cell; Table S4: TEAD family, Hippo effector, and canonical YAP/TAZ-TEAD target gene expression in highly migratory versus weakly migratory subpopulations; Figure S3: The prognostic association of the canonical TEAD4 target signature reflects tumor-type composition rather than signature activity; Table S5: Cohort definitions, hazard ratios, heterogeneity statistics, and expression correlations for all survival analyses; Figure S4: The EVA1A–TEAD4 expression relationship is cancer-type dependent and vanishes when tumor types are pooled.

## Abbreviations

BSA	Bovine serum albumin
CPTAC	Clinical Proteomic Tumor Analysis Consortium
DESeq2	Differential gene expression analysis tool
DMEM	Dulbecco’s Modified Eagle Medium
EMT	Epithelial–mesenchymal transition
FBS	Fetal bovine serum
FDR	False discovery rate
GO	Gene Ontology
GSEA	Gene set enrichment analysis
HM	Highly migratory
IPA	Ingenuity Pathway Analysis
ORA	Over-representation analysis
PBS	Phosphate-buffered saline
PFA	Paraformaldehyde
qPCR	Quantitative polymerase chain reaction
RNA-seq	RNA sequencing
WM	Weakly migratory
